# Flap Salvage Using Topical Oxygen Therapy (Natrox) in a Pediatric Foot Degloving Injury: A Case Report

**DOI:** 10.3390/jcm15134933

**Published:** 2026-06-25

**Authors:** Dong Wan Kim, Heui Ro Na, Seung Hyun Kim, Jun Ho Choi, Jae Ha Hwang, Kwang Seog Kim

**Affiliations:** Department of Plastic and Reconstructive Surgery, Chonnam National University Hospital, Chonnam National University Medical School, Gwangju 61469, Republic of Korea; waaan37@gmail.com (D.W.K.); skgmlfh1@naver.com (H.R.N.); hi_1004@naver.com (S.H.K.); cjh_0502@hanmail.net (J.H.C.); pskim@chonnam.ac.kr (K.S.K.)

**Keywords:** child, wounds and injuries, foot injuries, skin transplantation, topical oxygen therapy

## Abstract

**Background**: Foot degloving injuries are associated with extensive soft-tissue disruption, compromised perfusion, and a high risk of flap necrosis. Hyperbaric oxygen therapy (HBOT) is known to enhance tissue oxygenation and support flap survival; however, its application in pediatric patients may be limited due to poor cooperation, intolerance to chamber-based treatment, and limited accessibility. **Case Presentation**: A 7-year-old girl presented with a crush injury to the left foot after being run over by a vehicle, resulting in severe soft-tissue damage. Evaluation revealed a dorsal foot degloving injury, a proximal phalanx fracture of the great toe, and dislocations of the fourth proximal interphalangeal and fifth distal interphalangeal joints. Emergency surgery included open reduction, K-wire fixation, debridement, and artificial dermal grafting using Pelnac. On postoperative day 1, the flap showed signs of compromised perfusion. As HBOT was not feasible, topical oxygen therapy using Natrox was applied continuously for 17 days. Serial wound assessments demonstrated gradual improvement in flap viability. Although ischemic changes developed in the toes, necrosis remained superficial and was successfully managed with local debridement and dressings. Residual skin defects with partial necrosis were treated with split-thickness skin grafting, which healed without major complications. The patient resumed ambulation after splint removal. **Conclusions**: In pediatric patients with compromised flaps in whom HBOT is not feasible, topical oxygen therapy may serve as a practical adjunctive treatment option. Although its independent effect cannot be established in a single case, this report suggests its potential role in flap salvage and in limiting tissue necrosis.

## 1. Introduction

Degloving injuries of the foot are severe soft-tissue injuries in which the skin and subcutaneous tissue are extensively separated from the underlying structures. This disruption can impair perfusion, promote tissue hypoxia, and increase the risk of flap compromise or necrosis. For this reason, achieving durable wound coverage while preserving tissue viability remains a major reconstructive challenge. Oxygen is essential for several phases of wound healing, including fibroblast proliferation, collagen synthesis, angiogenesis, epithelial migration, and host defense against infection. When wounds become hypoxic, these oxygen-dependent processes are impaired, and healing may stall [1,2].

Hyperbaric oxygen therapy (HBOT) has long been used to improve tissue oxygenation in difficult wounds and compromised tissues. This therapy delivers 100% oxygen at increased atmospheric pressure, typically between 2 and 3 atmospheres, producing marked hyperoxemia and tissue hyperoxia. Under these conditions, plasma-dissolved oxygen increases substantially, thereby enhancing oxygen delivery to poorly perfused tissues and supporting multiple healing mechanisms [3]. Despite these potential benefits, HBOT requires specialized facilities, repeated chamber sessions, and adequate patient cooperation, all of which may limit its use in children. By contrast, topical oxygen therapy (TOT) delivers oxygen directly to the wound surface under normobaric conditions and can be used in inpatient, outpatient, or home settings. Consensus and review documents have described TOT as a practical adjunctive modality that may help reverse local hypoxia and support the enzymatic and cellular processes required for tissue repair [1,2].

Topical oxygen has been associated with improved local oxygen tension, enhanced angiogenic signaling, increased vascular endothelial growth factor (VEGF) expression, greater collagen deposition, and improved neovascularization. Experimental studies in ischemic wound models have further shown that topical oxygen application can accelerate wound healing, increase granulation tissue formation, and promote new-vessel formation [4,5].

Most clinical evidence for TOT has been generated in chronic wounds. Recent meta-analyses have reported higher healing rates and greater wound-area reduction when adjunctive TOT is added to standard care [6]. Evidence in acute traumatic wounds with compromised flaps is much more limited, and pediatric cases remain particularly scarce [7].

In this report, we describe a pediatric dorsal foot degloving injury with early postoperative flap compromise in which TOT using Natrox was selected because HBOT was not feasible. The favorable clinical course suggests that TOT may be considered as a practical adjunctive treatment in selected cases of flap compromise.

## 2. Case Presentation

**Figure 1:**
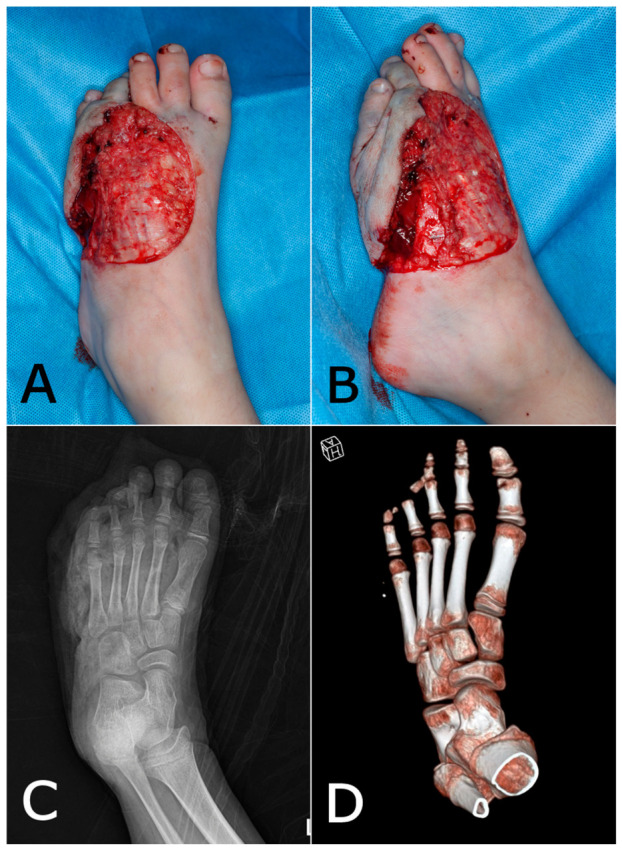
Preoperative clinical photographs, radiograph, and three-dimensional computed tomography image. (**A**,**B**) Preoperative clinical photographs demonstrating a degloving injury of the dorsal aspect of the left foot. (**C**,**D**) Preoperative radiograph and three-dimensional computed tomography image showing a fracture of the proximal phalanx of the great toe, with dislocation of the fourth proximal interphalangeal and fifth distal interphalangeal joints.

A 7-year-old girl was transferred to our institution after sustaining a crush injury to the left foot when the foot was run over by a vehicle. Initial evaluation revealed a dorsal foot degloving injury, a fracture of the proximal phalanx of the great toe, and dislocations of the fourth proximal interphalangeal and fifth distal interphalangeal joints (Figure 1). A concomitant anterior talofibular ligament injury was also suspected.

**Figure 2:**
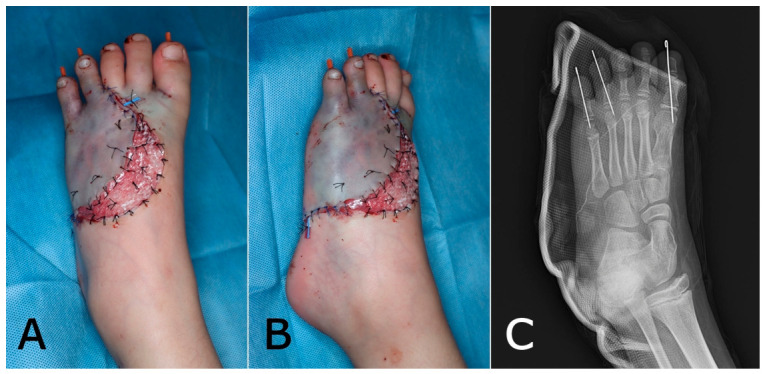
Immediate postoperative clinical photographs and radiograph. (**A**,**B**) Immediate postoperative clinical photographs after open reduction, K-wire fixation, debridement, and primary closure of the degloved flap, with artificial dermal grafting using Pelnac applied to the skin defect. (**C**) Postoperative radiograph confirming reduction and K-wire fixation.

Emergency surgery was performed and included open reduction, K-wire fixation, and debridement. The degloved skin flap was repositioned and closed primarily. At the distal portion of the flap, the tissue was extremely thin, with only a thin layer of skin remaining. Because the traumatic injury had also caused a partial skin defect, artificial dermal grafting with Pelnac was applied to the defect (Figure 2). The ankle ligament injury was treated conservatively with splint immobilization for 6 weeks.

On postoperative day 1, the flap showed dark discoloration, and pinprick testing yielded dark blood, indicating compromised perfusion. Both arterial insufficiency and venous congestion were suspected. However, because the flap was extremely thin and consisted almost entirely of skin, and because the degree of congestion was insufficient to require drainage, leech therapy was considered inappropriate. HBOT was also considered. However, because of the patient’s young age, concerns regarding cooperation and tolerance of repeated chamber-based treatment sessions limited its practical use. Although HBOT may be feasible in pediatric patients under sedation in specialized centers, repeated sedation was not considered desirable in this case. Therefore, TOT was selected as a practical and noninvasive alternative.

**Figure 3:**
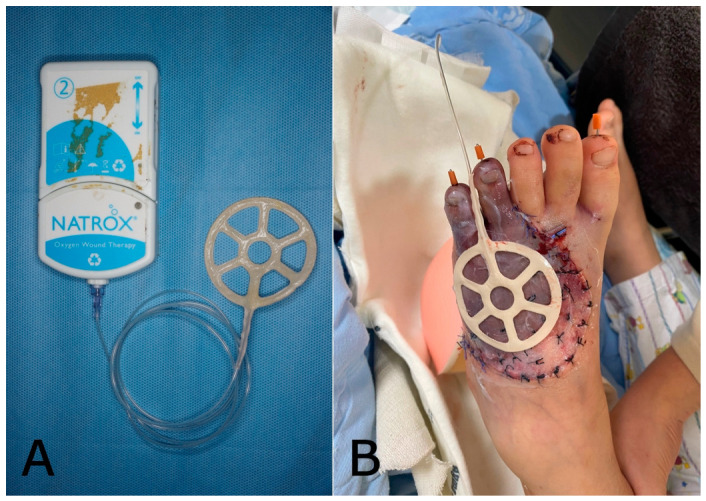
Application of topical oxygen therapy using Natrox. (**A**) The Natrox topical oxygen therapy device. (**B**) Application of the device to the wound. Humidified oxygen was delivered continuously through a fine tube to the dressing covering the flap.

**Figure 4:**
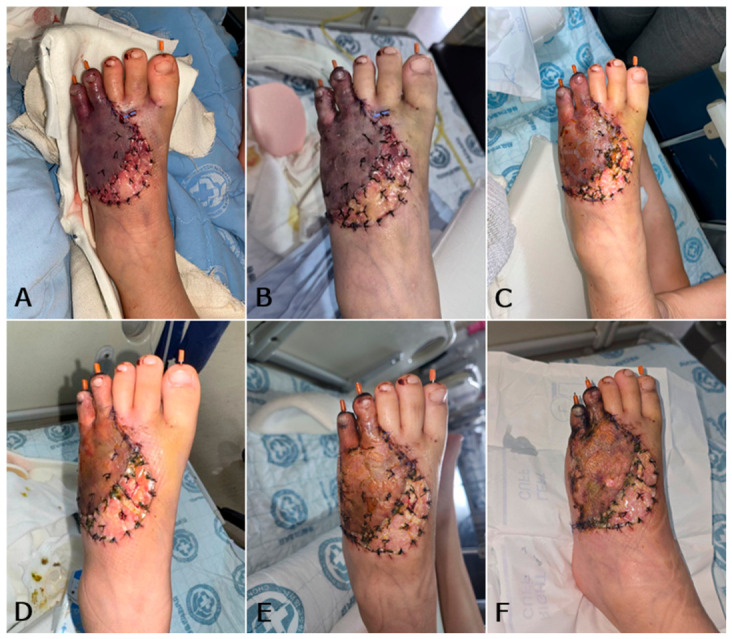
Serial clinical photographs during topical oxygen therapy. Serial postoperative photographs demonstrating changes in flap viability during 17 days of topical oxygen therapy. (**A**) Postoperative day (POD) 1, (**B**) POD 4, (**C**) POD 8, (**D**) POD 11, (**E**) POD 14, and (**F**) POD 17. Gradual improvement in flap color and progressive demarcation of viable tissue were observed over time.

TOT using Natrox was initiated immediately after wound dressing and was applied continuously (Figure 3). The wound was covered with gauze, and a heat lamp was applied as an additional supportive measure. Humidified oxygen was delivered continuously at 13 mL/h through a fine, soft tube connected to the device. Because the wound was located on the dorsum of the foot and extended toward the toes, maintaining stable positioning of the oxygen delivery system was challenging. The device was secured with secondary gauze dressings and protected from displacement during patient movement. Daily dressing changes allowed regular wound assessment while maintaining effective oxygen delivery. Dressings were changed daily. The flap color gradually improved during treatment, and TOT was discontinued after 17 days (Figure 4).

On postoperative day 19, the K-wire was removed. At postoperative week 5, split-thickness skin grafting was performed over the residual skin defect at the previous Pelnac-treated wound bed. The graft healed uneventfully, and no graft-related complications occurred.

**Figure 5:**
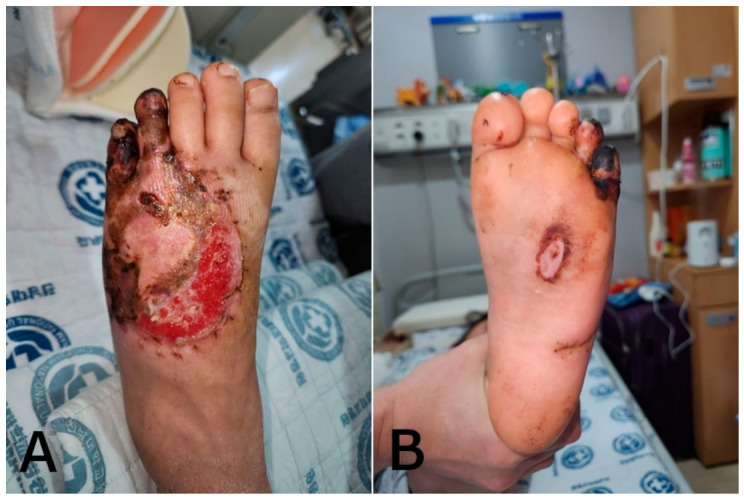
Clinical photographs obtained on POD 32 demonstrating superficial toe necrosis. (**A**,**B**) Clinical photographs demonstrating ischemic changes and superficial necrosis of the fourth and fifth toes. Necrosis was limited to the epidermal layer, without progression to deep-tissue involvement.

**Figure 6:**
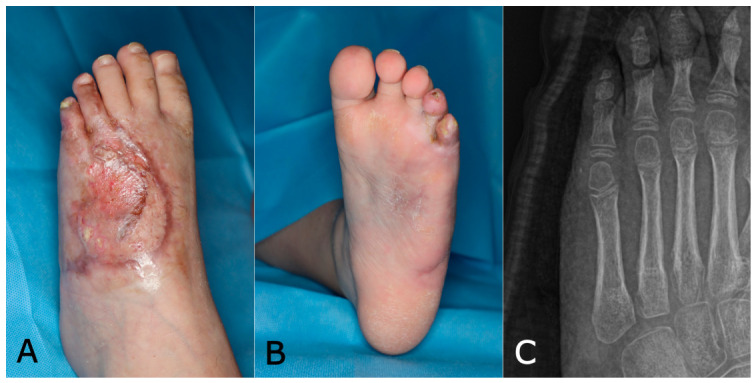
Clinical photographs and radiograph at follow-up. (**A**,**B**) Follow-up clinical photographs demonstrating preserved flap viability and stable wound healing, without significant scar contracture or deformity. (**C**) Follow-up radiograph demonstrating satisfactory alignment of the phalanges.

Progressive ischemic changes in the fourth and fifth toes were present from the time of injury and persisted throughout the postoperative course (Figure 5). Nevertheless, necrosis remained confined to the epidermal layer, without deep-tissue involvement, and healed with local debridement and dressings. After splint removal, the patient underwent rehabilitation and achieved successful ambulation. She continued regular outpatient follow-up after discharge (Figure 6). At the 6-month follow-up, no significant complications, such as restricted toe motion, impaired ambulation, or scar contracture, were observed.

## 3. Discussion

Several forms of topical oxygen therapy are currently available. Continuous diffusion oxygen therapy (CDOT), represented by systems such as Natrox, delivers a continuous low-flow supply of oxygen directly to the wound surface under normobaric conditions. In contrast, cyclically pressurized topical oxygen systems, such as TWO2, combine oxygen delivery with intermittent pressurization. Each modality has potential advantages and limitations regarding portability, treatment setting, patient compliance, and cost. In the present case, Natrox was selected because it allowed continuous bedside oxygen delivery without the need for specialized facilities.

Oxygen is fundamental to wound healing. It supports fibroblast activity, collagen deposition, angiogenesis, epithelialization, and oxidative bacterial killing. When tissue oxygen tension is insufficient, these processes become less efficient, and wounds are more likely to remain inflamed, poorly granulated, or infected. Accordingly, consensus and review papers consistently identify local hypoxia as a major barrier to healing and describe oxygen supplementation as a rational adjunctive strategy for difficult wounds [1,2].

The biological rationale for TOT is supported by mechanistic and experimental studies. Gordillo et al. reported that topical oxygen was associated with reduced chronic wound size and increased VEGF expression at the wound edge, suggesting a possible link between topical oxygen delivery and angiogenic signaling [4]. Animal studies have similarly shown that topical oxygen application can enhance wound healing, increase granulation tissue formation, and improve neovascularization [5].

Clinical evidence is strongest for chronic diabetic foot ulcers. A recent meta-analysis by Putri et al. found that adjunctive TOT significantly increased the number of healed wounds in randomized trials and observational studies and was associated with greater wound-area reduction [8]. Similarly, the diabetic foot ulcer meta-analysis by Sethi et al. found a higher complete-healing rate when TOT was added to standard care, while emphasizing that the overall certainty of evidence remained limited because several included studies had a moderate or high risk of bias [9]. Thus, the current literature supports TOT as a promising adjunct, but not as a definitive stand-alone therapy across all wound types. This balanced interpretation is also reflected in a recent scoping review, which concluded that the evidence is most convincing for chronic wounds and that broader applications require further study [2].

In clinical practice, HBOT is commonly used to enhance tissue oxygenation and support flap survival. However, its use in pediatric patients may be limited by poor cooperation, limited treatment accessibility, and difficulty tolerating chamber-based therapy. In these circumstances, TOT offers a practical, noninvasive bedside alternative.

Although described as flap compromise, the avulsed tissue in this case consisted of an extremely thin degloved skin flap with minimal subcutaneous tissue. Therefore, its biological behavior may partially resemble that of a skin graft. The therapeutic objective of topical oxygen therapy was to support survival of the compromised avulsed tissue while preserving as much viable coverage as possible. Accordingly, the observed tissue preservation may reflect effects on both flap-like tissue viability and graft-like skin survival.

Degloving injuries involving the toes are often accompanied by severe vascular compromise and carry a high risk of digital necrosis, which may lead to partial or complete amputation. Even after fracture-dislocation is reduced and stabilized appropriately, disruption or kinking of the neurovascular bundle may cause progressive ischemia and tissue loss. Previous reports of the “empty toe” phenomenon have shown that underlying vascular injury can lead to gangrene and poor outcomes despite apparently intact skin or successful reduction [10,11].

In the present case, the patient had several risk factors for digital loss, including a crush mechanism, degloving injury, toe fracture-dislocation, and K-wire fixation. Despite these risk factors, the fourth and fifth toes were preserved, and necrosis remained limited to the superficial epidermal layer. This outcome is notable because similar injuries have been reported to progress to deep necrosis or even autoamputation despite appropriate surgical management [11].

Although the independent effect of TOT cannot be definitively established from a single case, improved local oxygenation may have helped limit the extent of ischemic damage. The favorable outcome observed in this case cannot be attributed solely to TOT, as surgical debridement, fracture stabilization, flap repositioning, meticulous wound care, adjunctive heat lamp application, and natural healing processes may also have contributed to the final result. Nevertheless, TOT may have contributed not only to flap salvage but also to limiting tissue loss and facilitating successful toe preservation in this high-risk injury. Further studies are needed to clarify the independent role of TOT in acute traumatic flap compromise.

## 4. Conclusions

In pediatric patients with compromised flap viability for whom HBOT is not feasible, TOT may serve as a practical adjunctive treatment. Although its independent effect cannot be determined from a single case, this report highlights the potential role of TOT in flap salvage.

## Data Availability

No new data were created or analyzed in this study. Data sharing is not applicable to this article.

## Abbreviations

The following abbreviations are used in this manuscript:

HBOT	hyperbaric oxygen therapy
TOT	topical oxygen therapy
VEGF	vascular endothelial growth factor
